# A multiplex serological assay for the characterization of IgG immune response to SARS-CoV-2

**DOI:** 10.1371/journal.pone.0262311

**Published:** 2022-01-13

**Authors:** Etienne Brochot, Vianney Souplet, Pauline Follet, Pauline Ponthieu, Christophe Olivier, Gaël Even, Christophe Audebert, Rémi Malbec

**Affiliations:** 1 Department of Virology, Amiens University Medical Center, Amiens, France; 2 Agents Infectieux Résistance et Chimiothérapie Research Unit, Jules Verne University of Picardie, Amiens, France; 3 Innobiochips, Loos, France; 4 GD Biotech, Douai, France; Qatar University, QATAR

## Abstract

In the fight against SARS-COV-2, the development of serological assays based on different antigenic domains represent a versatile tool to get a comprehensive picture of the immune response or differentiate infection from vaccination beyond simple diagnosis. Here we use a combination of the Nucleoprotein (NP), the Spike 1 (S1) and Spike 2 (S2) subunits, and the receptor binding domain (RBD) and N-terminal domain (NTD) of the Spike antigens from the CoViDiag^®^ multiplex IgG assay, to follow the immune response to SARS-CoV-2 infection over a long time period and depending on disease severity. Using a panel of 209 sera collected from 61 patients up to eight months after infection, we observed that most patients develop an immune response against multiple viral epitope, but anti-S2 antibodies seemed to last longer. For all the tested IgGs, we have found higher responses for hospitalized patients than for non-hospitalized ones. Moreover the combination of the five different IgG responses increased the correlation to the neutralizing antibody titers than if considered individually. Multiplex immunoassays have the potential to improve diagnostic performances, especially for ancient infection or mild form of the disease presenting weaker antibody responses. Also the combined detection of anti-NP and anti-Spike-derived domains can be useful to differentiate vaccination from viral infection and accurately assess the antibody potential to neutralize the virus.

## 1. Introduction

Since its first detection in Wuhan (China) in December 2019, the Severe Acute Respiratory Syndrome Coronavirus 2 (SARS-CoV-2) has rapidly spread to reach other countries worldwide as the coronavirus 2019 disease (COVID-19) became pandemic [1].

The virion has a nucleocapsid composed by genomic RNA and phosphorylated Nucleocapsid (NP) protein, which is buried inside a phospholipid bilayer and covered by the Spike proteins trimmers (S) that gives the CoVs their crown-like appearance on which their names are based. The S protein has two subunits, the Spike 1 (S1) which contains the receptor-binding domain (RBD) and N-terminal domain (NTD) and the Spike 2 (S2) [2]. The choice of the antigenic domain is important, as it must be specific to the SARS-CoV-2 for discrimination against other hCoVs for example, and sensitive enough so infection would not be missed [3]. Also, anti-RBD antibodies are known to play a role in patients protection as this domain is used by the virus to penetrate host cells [4]. Most commercial serological assays have demonstrated satisfying performances in terms of diagnostic sensitivity and specificity, based on one of those main different antigenic domains [5, 6]. However, the combination of different immunogenic antigens can give a more comprehensive picture of the humoral response strength and diversity [7–9] while maintaining elevated diagnostic performances [10, 11]. In multiplex assays, positivity thresholds can be adjusted to compensate for the use of antigenic domains more conserved between coronaviruses [12]. Moreover, as vaccines are based on the Spike protein, the additional detection of anti-NP antibodies allows to differentiate viral infection from vaccination.

This study reports the use of the CoViDiag^®^ multiplex IgG assay for the characterization of the immune response against over time, depending on disease severity, and in perspective of neutralizing antibody titers.

## 2. Material and methods

### 2.1. Study design and cohort

The study was conducted at Amiens University medical Center (France). Samples were derived from de-identified excess serum specimens. The demographic information of the patients are available in Table 1. The study was approved by the institutional review board of the Amiens University Medical Center (number PI2020_843_0046, 21 April 2020).

**Table 1:** Cohort characteristics.

Number of patients	61
Total number of samples collected	209
Number of samples/patient:	
Median	3
Range	1–8
Female	36
Male	25
Age (Years):	
Median	74
Range	26–98
>65 years	41
Hospitalized patients	27
Nonhospitalized patients	34
Immunocompromised patients	6 (2 kidney transplant, 2 bone marrow transplant, 2 chemotherapy)
Number of samples (days post-PCR)	
0–59	52
60–119	49
120–179	49
≥180	59
Number of patients (days post-PCR)	
0–59	50
60–119	36
120–179	42
≥180	46

Briefly, we used n = 209 samples collected between March and April 2020 from n = 61 patients (27 hospitalized patients and 34 non-hospitalized patients) with PCR-confirmed SARS-CoV-2 infections to perform immunoassay and virus seroneutralization test as already described in Aubry et al. [13]. All samples have been tested according to manufacturer’s instruction on the CoViDiag^®^ serological assay and the raw results are available in supplementary data.

### 2.2. Serological assay

**Fig 1:**
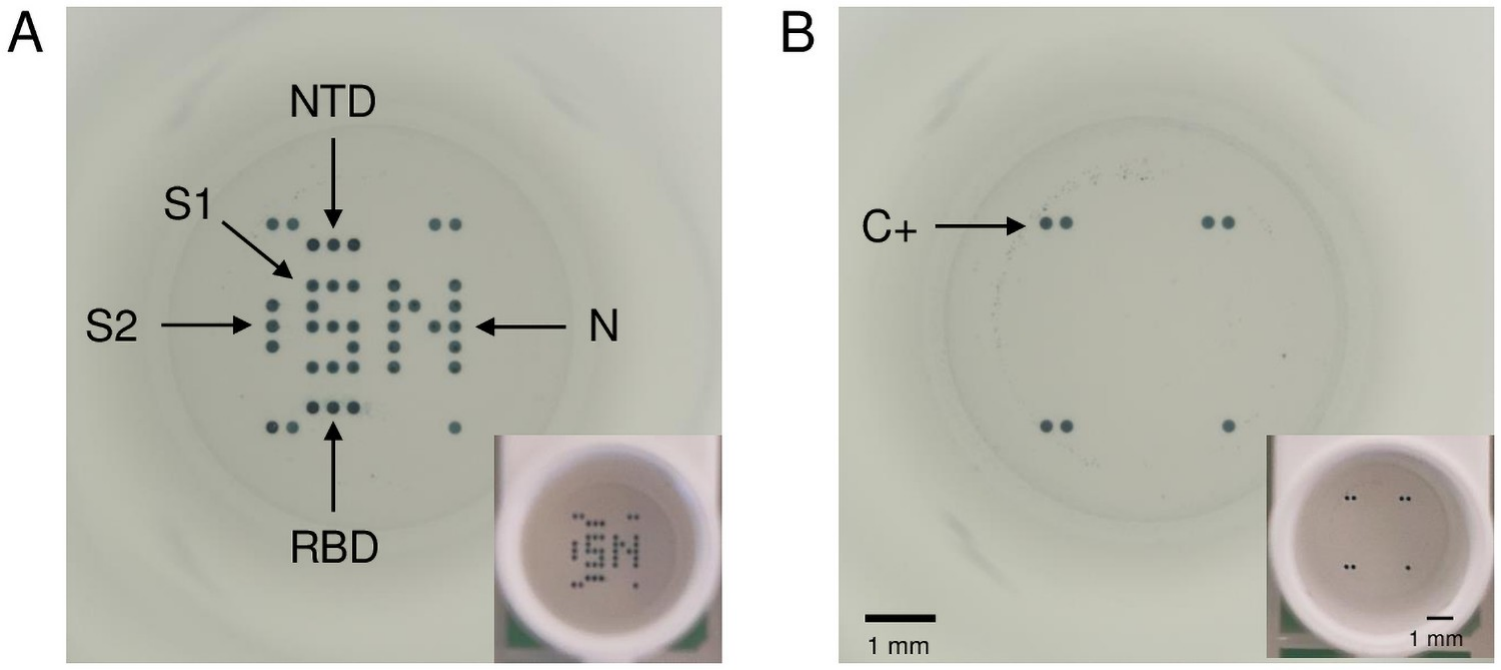
Full well pictures pictures obtained with the microplate reader (SciReader^®^) or with a phone camera (in insert) after incubation with the CoViDiag^®^ assay. (A) Positive sample presenting antibodies against the Nucleopcapside (NP), Spike 1 (S1), N-terminal domain (NTD) and Receptor binding domain (RBD) of the Spike protein, or Spike 2 (S2) antigens. (B) Negative sample with positive control on the edges. Scale bars correspond to 1 mm.

The CoViDiag^®^ multiplex immunoassay is based on the ELISA principle and targets IgG antibodies against five different antigens of the SARS-CoV-2 virus: NP, S1, S2, RBD, and NTD (Fig 1). Note that the S1 and NP antigens have been printed in dot replicates in the shape of an “S” and “N” letters, respectively. This design allows for quick visual interpretation of seropositivity and vaccination status according to the manufacturer’s instruction (IFU in S1 File).

Briefly, serum samples (100 μL per well) were diluted 1:100 in the provided ready to use Diluent Buffer. The plates were incubated 1 h at 37 °C on a microplate shaker at 300 rpm, and washed three times (200 μL/well) with the provided Washing Buffer. 60 μL of Conjugate Antibody was added to 10 mL of Dilution Buffer for conjugation and 100 μL of diluted conjugate was added to each well, followed by 1 h incubation at 37 °C in the dark. After washing, 50 μL of provided Substrate solution was added to each well and incubated for 15 min in the dark. After a final washing step with 200 μL of mQ water per well, any trace of residual water was removed by incubation for 15 min at 37 °C. Distinguishable individual spot (circular “blue dots”) are visible at the surface of the wells when IgG antibodies have been specifically captured by the corresponding antigens. The color intensity is correlated to the amount of antibodies present in the sample. Images of individual wells were captured by a microplate reader (SciReader^®^, Scenion GmbH) and associated software for spot detection and spot intensity measurement. The spot mean signal intensity (MSI) in arbitrary unit (a.u.) was calculated as the average pixel value inside the spot perimeter minus the local background around the spot as described in Malbec et al. [14]. For automatic delivering of the diagnosis results, an algorithm combining different cut-off (reported in Table 2) has been set in the software as recommended by the CoViDiag^®^ Instruction For Use (see IFU section 8.4 in S1 File).

**Table 2:** Mean signal intensity cut-offs for individual antigens in arbitrary units (a.u.).

Antigen	Negative	Borderline	Positive
NP	0–15	15–30	> 30
S1	0–10	10–20	> 20
RBD	0–10	10–20	> 20
NTD	0–10	10–20	> 20
S2	0–15	15–30	> 30

Samples are identified as IgG positive to SARS-CoV-2 when S1 and/or RBD and/or NTD is positive, or S2 and/or NP MSI is > 40 a.u, or S2 and/or NP is positive and S1 and/or RBD and/or NTD and/or S2 and/or NP is borderline, or S2 and/or NP is borderline and S1 and/or RBD and/or NTD and/or S2 and/or N is borderline.

### 2.3. Neutralization assay

Retroviral particles pseudotyped with the S glycoprotein of SARS-CoV-2 (SARS-CoV-2pp) were produced, with a plasmid encoding a human codonoptimized sequence of the SARS-CoV-2 spike glycoprotein (accession number: MN908947), as previously described in Brochot et al. [3]. Supernatants containing the pseudotyped particles were harvested at 48, 72, and 96 h after transfection, pooled, and filtered through 0.45-μm pore-sized membranes. Neutralization assays were performed by preincubating SARS-CoV-2pp and serially diluted plasma for 1 h at room temperature before contact with 293T cells (ATCC^®^ CRL-3216TM) transiently transfected with the plasmids pcDNA3.1-hACE2 24 h before inoculation. Luciferase activity was measured 72 h postinfection, as indicated by the manufacturer (Promega). Two independent tests were carried out each time in duplicate. The NAb titers were defined as the highest dilution of plasma resulting in a 90% decrease in infectivity. We previously controlled the specificity of our neutralization assay using not only plasmas from patients seropositive for other coronaviruses but also retroviral particles pseudotyped with the G glycoprotein of the vesicular stomatitis virus.

### 2.4. Statistical analysis

For the statistical analysis, Student’s test was used to test the relationship between different categorical variables and the difference in antibody MSI between hospitalized and non-hospitalized groups of patients. Spearman’s rank Correlation test was used to test the correlation between different antibody MSI and dilution factor for the neutralization assay. The general significance level was set at a p-value below 0.05. All analyses were performed using packages stats from the R statistical computing program v. 3.6.1 (Date of release 07/05/2019).

## 3. Results

### 3.1. Evolution of the IgG profile over time

**Fig 2:**
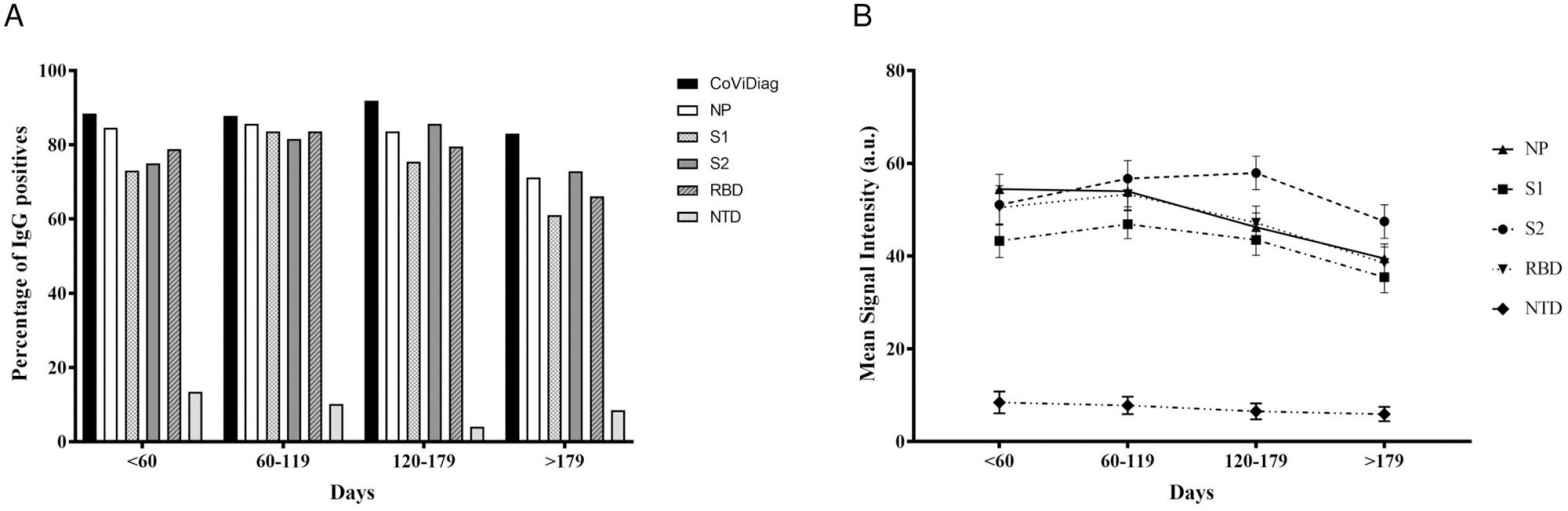
Evolution of the IgG profile over time. (A) Percentage of patients CoViDiag^®^ positive to anti-NP, anti-S1, anti-S2, anti-RBD, and anti-NTD IgG antibodies and (B) associated average spot intensity (MSI) of IgG responses expressed as arbitrary units (a.u.) over time.

Using the CoViDiag^®^ assay on 209 serum samples, we have observed over 87% seropositivity up to 6 months after an initial positive SARS-CoV-2 RT–PCR, before decreasing to 83% between six and eight months, as the seropositivity of individual IgGs targeting different antigens starts to drop (Fig 2A). As the overall IgG response gets weaker in time, the combined detection of IgGs to different antigenic domain allow to maintain elevated diagnostic sensitivity.

Positivities for each IgG considered individually are also reported based on the cut-offs set by the manufacturer (Table 2). More than a half of the samples (54.1%, n = 113/209) were concomitantly positives for anti-NP, anti-S1, anti-S2 and anti-RBD antibodies and 9.1% (n = 19/209) for all 5 antibodies. This result show that infected people generally develop antibodies against a wide spectra of the virus immunogenic domains. However, 4.3% (n = 9/209) samples (n = 6 for anti-NP, n = 2 for anti-S1 and n = 1 for anti-S2) presented a single positive antibody against the tested immunogenic domains (Table 3). The combination of multiple antigens may then help to slightly increase diagnostic sensitivity.

**Table 3:** Prevalence of the profile of IgG immune response. Number of samples with antibodies targeting single or combination of antigenic domains.

	N	% Seroprevalence
Samples	209	
Single antibody		
*NP*	6	2.9
*S1*	2	1.0
*S2*	1	0.5
*RBD*	0	0
*NTD*	0	0
*Total*	9	4.3
Combinaton of antibodies*		
*NP+S2*	5	2.4
*NP+S1+S2*	5	2.4
*NP + S1 + RBD*	6	2.9
*NP+S2+RBD*	13	6.2
*S1+S2+RBD*	6	2.9
*NP+S1+S2+RBD*	113	54.1
*NP+S1+S2+RBD+NTD*	19	9.1

* For clarity purpose, only most common combinations with >2% seroprevalence are reported.

The kinetics of the IgG serum antibody response to individual antigens are presented in Fig 2B. Average MSIs have been calculated for all samples depending on the time post RT-PCR to SARS-CoV-2. The anti-NP and anti-NTD antibody responses were the first to decrease, as their MSI started to decline after just two months (-0.9% and -8.1% between two and four months, respectively). The anti-S1 and anti-RBD response peaked after four months, before significatively decreasing over time (-7.8% and -13% between four and six months, respectively). The anti-S2 antibody response was the most delayed, with a peak level reached between four and six months. The different dynamics observed are in accordance with the combination of multi-antigens at different time point. In the first two months after a positive RT-PCR to SARS-CoV-2, an IgG response to a single antigen is observed in 5.8% (n = 3/52) of the samples and a concomitant IgG response to NP, S1, S2, and RBD antigens is observed in 51.9% (n = 27/52) of the samples (S1 Table). Between two and six months, the increase of the MSI measured for the different IgGs correlates with a diversification of the IgG response, as the IgG response to a single antigen is only observed in 2% (n = 2/98) of the samples while the frequency of observation of concomitant IgG response to NP, S1, S2, and RBD antigens increases to 61.2% (n = 60/98) of the samples. However after six months, the diversity of the IgG response decrease with the measured MSI, and IgG response to a single antigen is observed in 6.8% (n = 4/59) of the samples while the frequency of observation of concomitant IgG response to NP, S1, S2, and RBD antigens drops to 39% (n = 23/59) of the samples.

Those results show the interest of detecting IgG response against multiple immunogenic domains to maintain elevated diagnostic sensitivity, especially long after infection.

### 3.2. IgG profile depending on the disease severity

**Fig 3:**
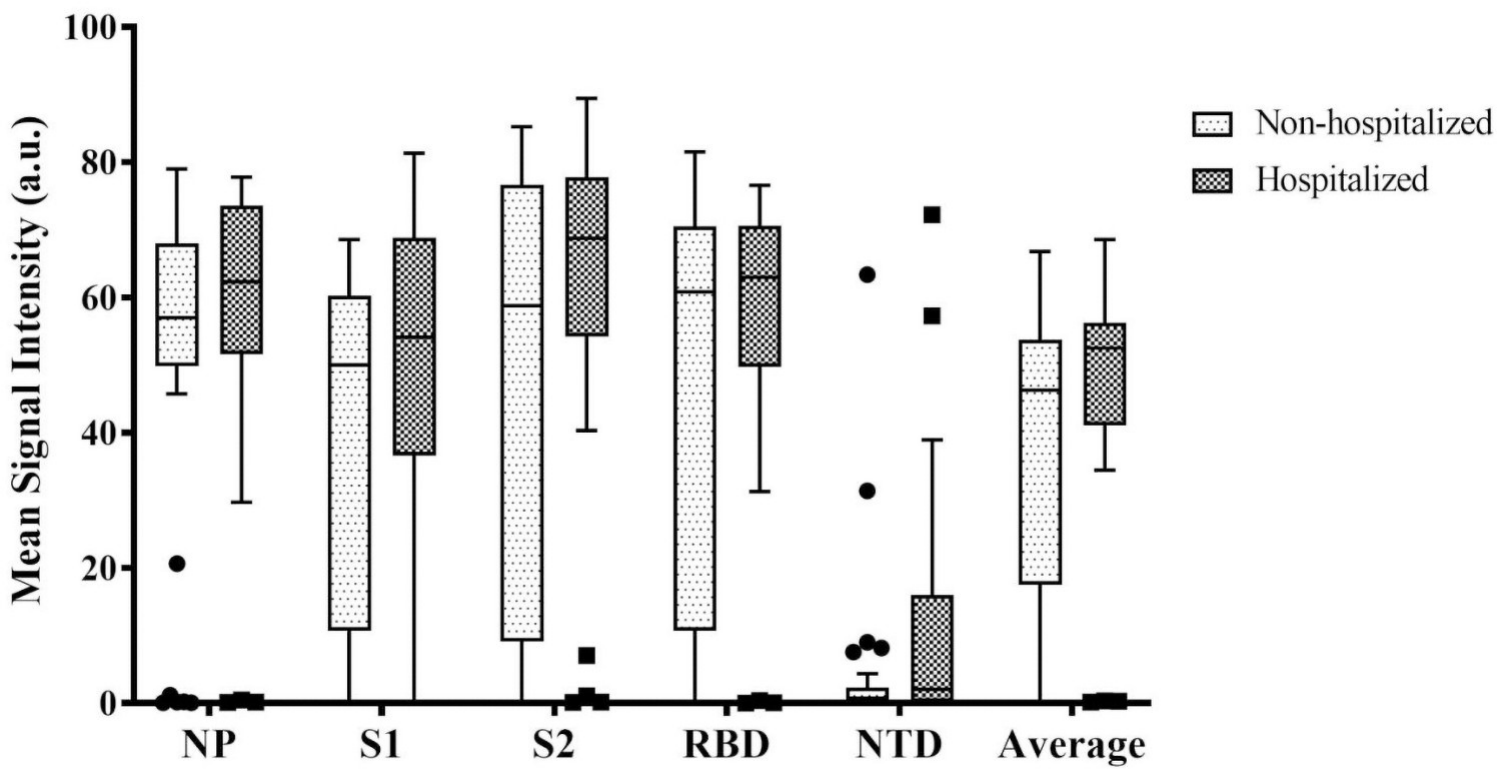
IgG profile depending on disease severity outcome. Distribution of the different IgG responses based on the MSI in arbitrary units (a.u.), considered individually, or altogether (average) for hospitalized (n = 25) and non-hospitalized patients (n = 34) just after infection.

Then we have investigated the ability for the multiplex assay to differentiate hospitalized (severe cases) versus non hospitalized (mild cases) patients, based on the first sample collected for each of the 61 patients in the early convalescent phase of the disease. For all five immunogenic domains, the MSI, corresponding to the levels of antibody are plotted in Fig 3, depending on disease severity. For each given antigen, we have observed a trend of greater antibody response for hospitalized patients (MSI: NP = 56.5 a.u.; S1 = 49.1 a.u.; S2 = 59.4 a.u.; RBD = 54.8 a.u.; NTD = 11.8 a.u.; Average = 46.3 a.u.) compared to non-hospitalized ones (MSI: NP = 51.8 a.u.; S1 = 37.4 a.u.; S2 = 49.2 a.u.; RBD = 47.1 a.u.; NTD = 4.3 a.u.; Average = 37.9 a.u.). However, the differences were not statistically different (p-value > 0.05, S2 Table).

### 3.3. Correlation between IgG profiles and neutralizing antibody titers

**Fig 4:**
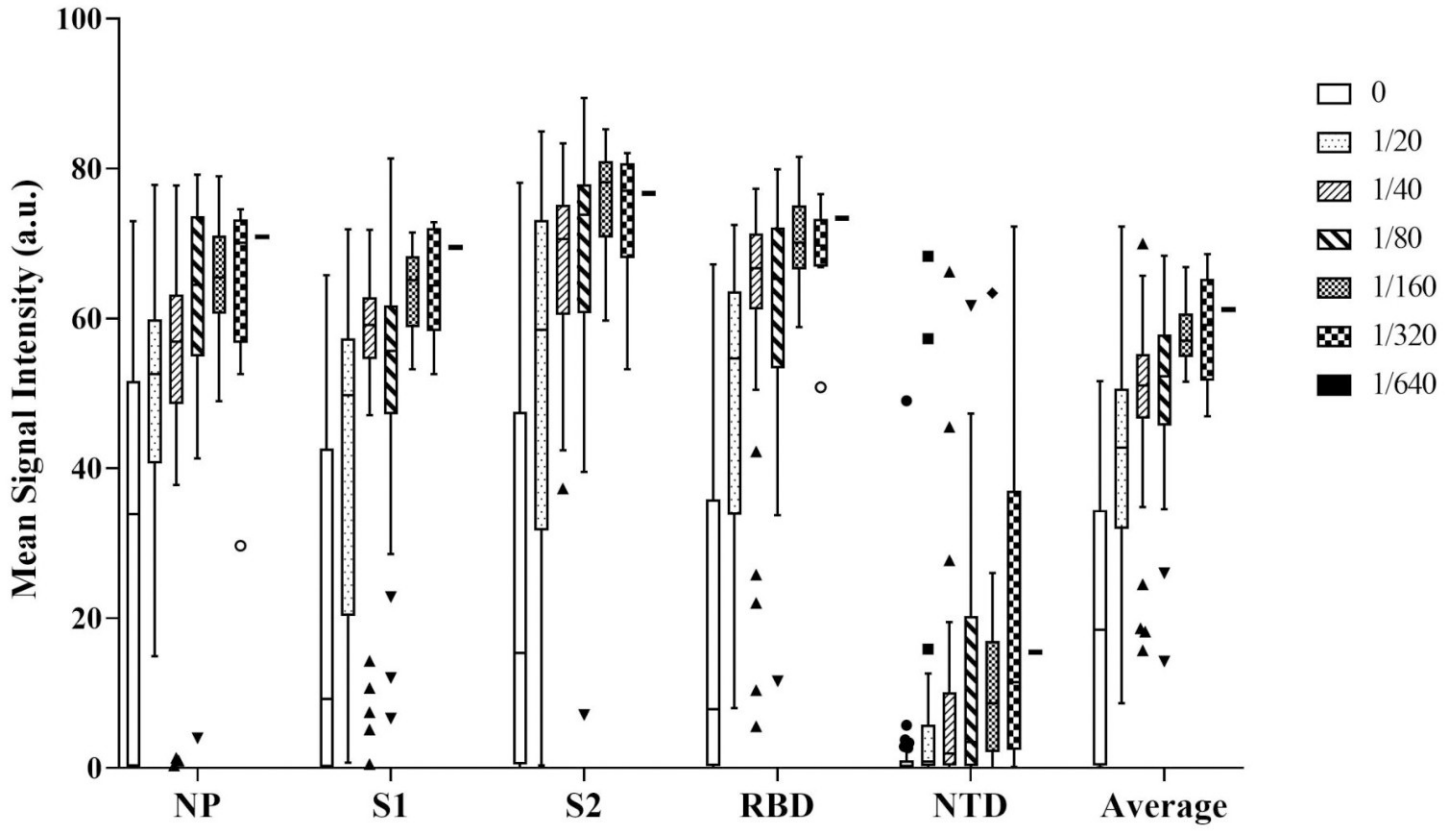
Correlation of the different IgG responses with serum neutralization titers. IgGs responses are based on the MSI in arbitrary units (a.u.) considered individually, or altogether (average). Neutralizing antibody titers are based on the serum dilution factor to neutralize 90% of infected cells.

Finally, we have evaluated the ability for the correlation between the different IgG levels response and the seroneutralization potential of the samples. For all five immunogenic domains, the mean intensity, corresponding to the levels of antibody response are plotted in Fig 4 depending on the highest dilution of serum resulting in a 90% decrease in infectivity. As expected, the best correlation (see S3 Table) between individual IgGs and neutralizing antibody response was obtained for anti-RBD antibodies (r^2^ = 0.72, p-value < 2.2e-16). The correlation was very similar between anti-S1 (r^2^ = 0.67, p-value < 2.2e-16) and anti-S2 (r^2^ = 0.66, p-value < 2.2e-16) antibodies. However Anti-NP (r^2^ = 0.59, p-value < 2.2e-16) and anti-NTD (r^2^ = 0.47, p-value = 3.813e-13) antibodies MSI were less correlated with the neutralizing antibody titers. Interestingly, the combination of the 5 different antibody responses, allowed to slightly increase the correlation to (r^2^ = 0.74).

## 4. Discussion

Several studies have found improved performances from use of antigen combinations that include both spike protein and Nucleoprotein [10, 15, 16]. Gillot et al. evaluated the CoviDiag^®^ assay and concluded that the combination of several antigens in the same test improves the overall specificity and sensitivity of the test [17]. Similarly, in our previous work based on the same set of sample, we have found equivalent to improved diagnostic performances, especially for ancient infections, for the CoViDiag^®^ multiplex IgG assay compared to other simplex IgG commercial assays [13]. Is is now generally admitted that antibody levels are weaker for asymptomatic and mild form of the disease and can decrease over time. For instance, Grossberg et al., have observed a more robust IgG response in positive/symptomatic participants than in positive/asymptomatic participants [8]. They were able to differentiate between severe, mild and asymptomatic group of participants using S1-RBD IgA, NP IgG and S2 IgA titers. Hence in the present work, we have investigated the profile of the IgG immune response over an eight months period with a multiplex assay, using samples of hospitalized and non-hospitalized patients. Then we have compared the results with neutralizing antibody levels.

We have observed that most patients develop a global immune response against multiple immunogenic domains. Even over an eight months period, more than a half of the samples were positives to anti-NP, anti-S1, anti-S2, and anti-RBD antibodies, concomitantly. Those result confirm the possibility to develop serological assays based on different antigens. Anti-NTD antibodies are more scarce. Using the multiplex technology from Meso Scale Diagnostics, LLC, Chaudhury et al, have also observed that IgM and IgG antibodies were less reactive to NTD than NP or RBD antigens [18]. One explanation might come from the fact that this domain shows the lowest sequence identity compared to SARS-CoV Spike protein. So the IgG response to NTD antigen may be more naïve than for others, resulting in decrease sensitivity but increase specificity potential for diagnostics, which was the initial reason for its presence in the CoViDiag^®^ multiplex assay. Also, as most SARS-CoV-2 infected patients develop antibodies against the NP antigen differentiation of infection from vaccination may be possible based on this antigen as vaccines are based on the Spike protein.

As expected, the different IgGs responses decreased over time, but with different dynamics. As the overall IgG response gets weaker in time, the probability of detecting an IgG response to a single antigen increases. Hence, the detection of IgG response to different antigenic domain may allow to maintain elevated diagnostic sensitivity. The evolution of anti-S1 and anti-RBD responses is very similar, as RBD constitutes a domain of the Spike 1 protein. However, elevated levels of anti-S2 IgG seem to last longer. Therefore the detection of anti-S2 IgG may be of interest to maintain elevated diagnostic sensitivity longer after infection. However as the S2 domain is highly conserved among coronavirus, its presence may not be very specific to SARS-CoV-2 infection. The CoViDiag assay algorithm adapts the cut-offs depending on the number of different IgGs detected to deliver SARS-CoV-2 positivity status, and maintain diagnostic sensitivity and specificity performances over time. Those results may explain our previous observations on the same cohort [13], where we have observed that the CoViDiag^®^ diagnostic sensitivity performance remained more stable over time than for two other commercial references of simplex IgG immunoassay (Abbot^®^ and Euroimmun^®^ IgG assays, based on the NP and the S1 antigen, respectively).

For all the tested IgGs, we have found higher MSI for hospitalized patients than for non-hospitalized ones. However, the differences were not statistically significant as a large number of patients had no immune response detected for individual antigens, independently of the disease severity. Those results are in accordance with the finding of Gillot et al. using the CoViDiag^®^ assay, who have observed a trend of higher signals for NP, S1, S2 and RBD antibodies from 14 days since symptom onset in critical patients, even if the differences were not statistically different compared to non-critical patients in their cohort [17]. It is noteworthy that most commercial assays outstanding performances have been established at the beginning of the epidemic, on samples with severe form of the disease, and possible strong immune response, as samples from hospitalized patients were the easier to collect. For people presenting a weaker immune response, multiplexing allows to test for extra domains that may help to slightly increase diagnostic sensitivity without compromising for diagnostic specificity.

Except for anti-NTD antibodies, all different IgGs MSI were positively correlated with the neutralizing antibody titers. This result is not surprising considering our previous observation showing that anti-NP, anti-S1, anti-S2, and anti-RBD antibodies are concomitantly present in patient’s sera. As expected, the best correlation for individual antigen is obtained for antibodies targeting the virus RBD domain which is known to be involved in the penetration of the cells by the virus. However the average combination of all five antigens slightly increased the correlation, strengthening the interest for multiplexing.

Even if testing for IgGs seem more appropriate for the evaluation of an efficient and long lasting protection of the patients, the restriction to this particular isotype is a limitation to this study. Several commercial assays have shown good performances focusing on the detection of total antibodies (IgG, IgM and IgA). Evaluating the IgA and IgM profile in multiplex would be of interest for future experiments. Future studies could also include the collection of samples with more uniform number and duration, the determination of antibody titers using calibration curves, and investigate the immune profile between more diverse forms of the disease as asymptomatic forms.

However the present work contributes to provide insights into the dynamic and diversity of the immune response over time and depending on the disease severity. Our results confirms those of previous study on the potential for multiplexing to improve diagnostic performances of COVID-19 serology assays.

## 5. Conclusion

Beyond the diagnosis of SARS-COV-2 infection, tools delivering a global picture of the patients’ immune response may also be of interest to improve the management and care of the patients and populations. Our results show that elevated IgGs responses against multiple viral epitope may be more characteristic of symptomatic patients, and correlates well with neutralizing antibodies. We recommend using assays targeting IgGs for the evaluation of a long lasting population protection and collective immunity. Furthermore, multiplexed assays have the potential to slightly increase diagnostic performances, especially for ancient or weak infections and be more representative of immune protection. For future epidemical studies, as the vaccination based on the Spike protein progresses, multiplex serological assays may also help to differentiate vaccination from viral infection and the immune response to different variants.

## Supporting information

S1 Table Prevalence of the profile of IgG immune response. Percentage of positivity for antibodies against different antigens or combination of antigens for different time period after RT-PCR positive to SARS-CoV-2.
(PPTX)

S2 Table Mean Signal Intensity (MSI) in arbitrary units (a.u.) of the antibody response to different antigens for hospitalized and non-hospitalized patients. (PPTX)

S3 Table Correlation between individual antigen responses and neutralizing antibody titers. (PPTX)

S1 File Instruction for use version 1.4 of the CoViDiag^®^ assay. (PDF)
