# Supplementary material for: A multiplex serological assay for the characterization of IgG immune response to SARS-CoV-2
Source: PLoS One. 2022 Jan 13;17(1):e0262311. doi: 10.1371/journal.pone.0262311 (PMC8757954; doi:10.1371/journal.pone.0262311)
Supplement: S1 Table — Percentage of positivity for antibodies against different antigens or combination of antigens for different time period after RT-PCR positive to SARS-CoV-2. (PPTX) [file pone.0262311.s001.pptx]

## Slide 1
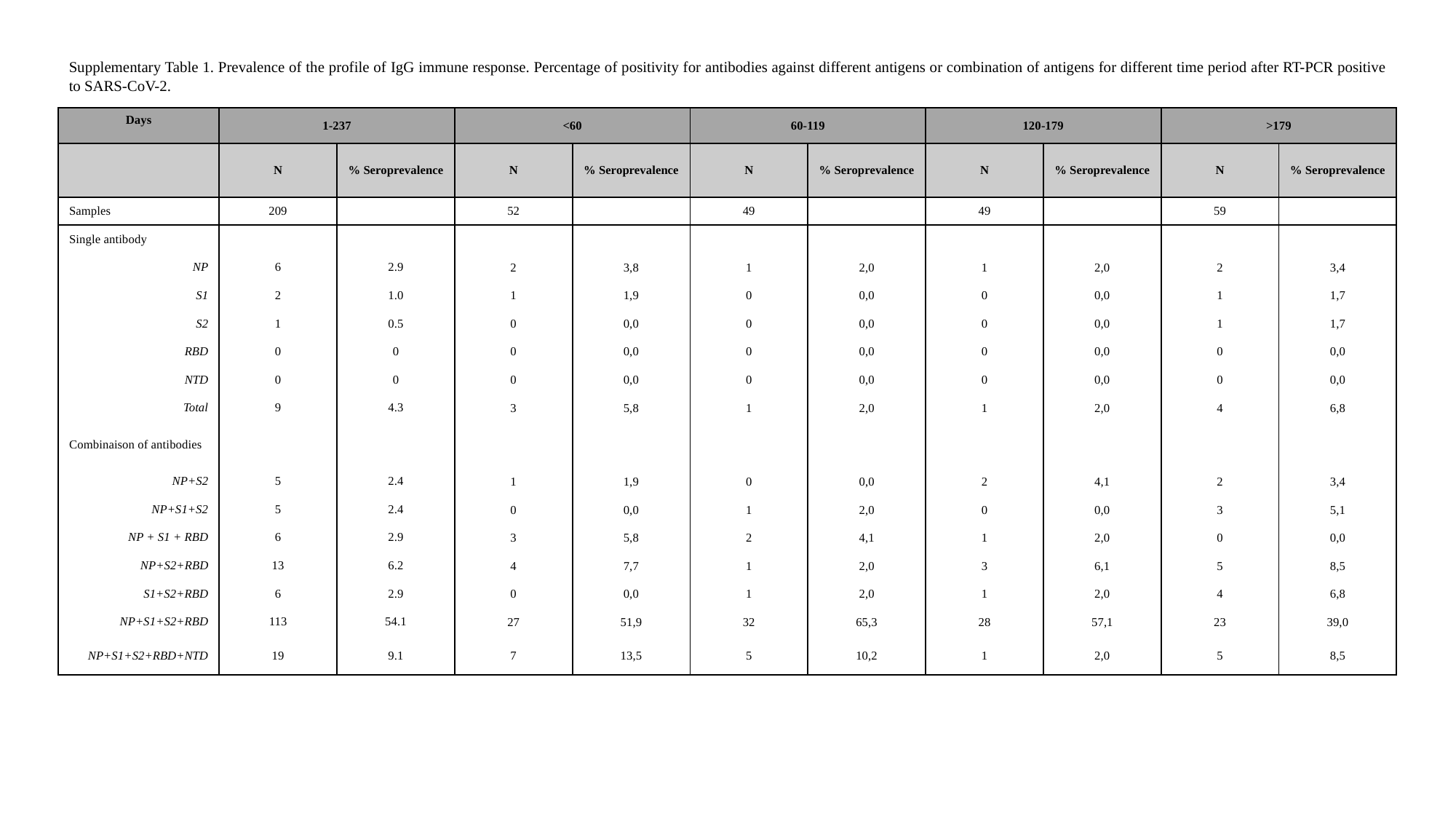

Supplementary Table 1. Prevalence of the profile of IgG immune response. Percentage of positivity for antibodies against different antigens or combination of antigens for different time period after RT-PCR positive to SARS-CoV-2.
| Days | 1-237 | | <60 | | 60-119 | | 120-179 | | >179 | |
| --- | --- | --- | --- | --- | --- | --- | --- | --- | --- | --- |
| | N | % Seroprevalence | N | % Seroprevalence | N | % Seroprevalence | N | % Seroprevalence | N | % Seroprevalence |
| Samples | 209 | | 52 | | 49 | | 49 | | 59 | |
| Single antibody | | | | | | | | | | |
| NP | 6 | 2.9 | 2 | 3,8 | 1 | 2,0 | 1 | 2,0 | 2 | 3,4 |
| S1 | 2 | 1.0 | 1 | 1,9 | 0 | 0,0 | 0 | 0,0 | 1 | 1,7 |
| S2 | 1 | 0.5 | 0 | 0,0 | 0 | 0,0 | 0 | 0,0 | 1 | 1,7 |
| RBD | 0 | 0 | 0 | 0,0 | 0 | 0,0 | 0 | 0,0 | 0 | 0,0 |
| NTD | 0 | 0 | 0 | 0,0 | 0 | 0,0 | 0 | 0,0 | 0 | 0,0 |
| Total | 9 | 4.3 | 3 | 5,8 | 1 | 2,0 | 1 | 2,0 | 4 | 6,8 |
| Combinaison of antibodies | | | | | | | | | | |
| NP+S2 | 5 | 2.4 | 1 | 1,9 | 0 | 0,0 | 2 | 4,1 | 2 | 3,4 |
| NP+S1+S2 | 5 | 2.4 | 0 | 0,0 | 1 | 2,0 | 0 | 0,0 | 3 | 5,1 |
| NP + S1 + RBD | 6 | 2.9 | 3 | 5,8 | 2 | 4,1 | 1 | 2,0 | 0 | 0,0 |
| NP+S2+RBD | 13 | 6.2 | 4 | 7,7 | 1 | 2,0 | 3 | 6,1 | 5 | 8,5 |
| S1+S2+RBD | 6 | 2.9 | 0 | 0,0 | 1 | 2,0 | 1 | 2,0 | 4 | 6,8 |
| NP+S1+S2+RBD | 113 | 54.1 | 27 | 51,9 | 32 | 65,3 | 28 | 57,1 | 23 | 39,0 |
| NP+S1+S2+RBD+NTD | 19 | 9.1 | 7 | 13,5 | 5 | 10,2 | 1 | 2,0 | 5 | 8,5 |
