# Supplementary material for: A multiplex serological assay for the characterization of IgG immune response to SARS-CoV-2
Source: PLoS One. 2022 Jan 13;17(1):e0262311. doi: 10.1371/journal.pone.0262311 (PMC8757954; doi:10.1371/journal.pone.0262311)
Supplement: S2 Table — (PPTX) [file pone.0262311.s002.pptx]

## Slide 1
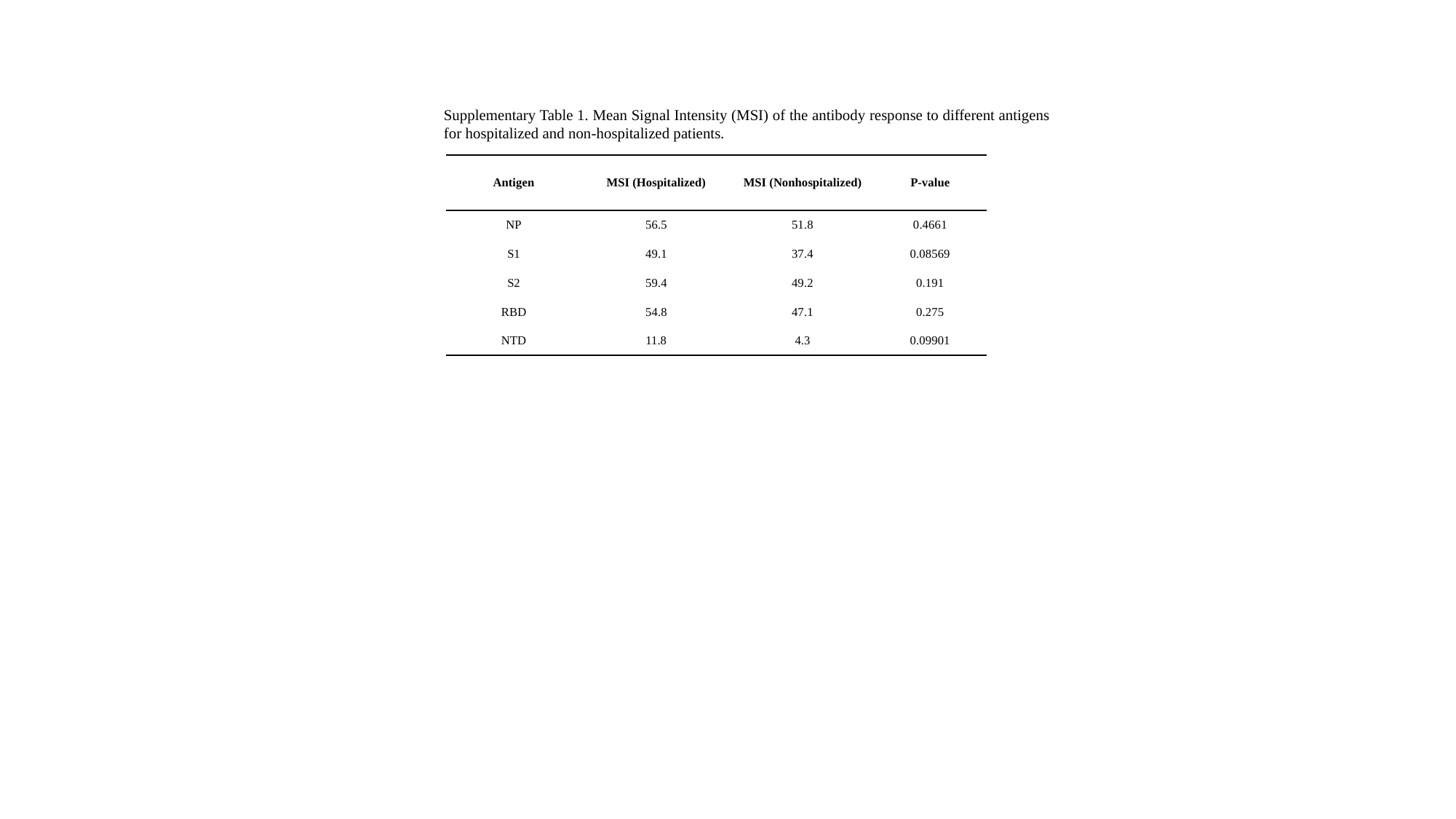

Supplementary Table 1. Mean Signal Intensity (MSI) of the antibody response to different antigens for hospitalized and non-hospitalized patients.
| Antigen | MSI (Hospitalized) | MSI (Nonhospitalized) | P-value |
| --- | --- | --- | --- |
| NP | 56.5 | 51.8 | 0.4661 |
| S1 | 49.1 | 37.4 | 0.08569 |
| S2 | 59.4 | 49.2 | 0.191 |
| RBD | 54.8 | 47.1 | 0.275 |
| NTD | 11.8 | 4.3 | 0.09901 |
