# Supplementary material for: A multiplex serological assay for the characterization of IgG immune response to SARS-CoV-2
Source: PLoS One. 2022 Jan 13;17(1):e0262311. doi: 10.1371/journal.pone.0262311 (PMC8757954; doi:10.1371/journal.pone.0262311)
Supplement: S1 File — (PDF) [file pone.0262311.s004.pdf]

Understanding life.
innobiochips

Instruction For Use

SirYus CoViDiag®

In-vitro diagnostic medical device for the anti-SARS-CoV-2 antibodies detection

IVD

REF 2001

CE

Version: 1.4
Issue: 2021-01

Table of content:

1. INTENDED USE 2
2. CONTEXT 2
3. PRINCIPLE OF THE TEST 3
4. MATERIAL 4
4.1 Reagents 4
4.2 Materials and equipment required 4
4.3 Optional materials and equipment 4
5. STABILITY AND STORAGE 4
6. REAGENTS PREPARATION 5
6.1 Test well strips 5
6.2 Washing buffer 5
6.3 Conjugate 5
6.4 Substrate solution 5
7. SAMPLES COLLECTION AND PREPARATION 6
7.1 Samples 6
7.2 Sample dilution 6
8. PROCEDURE OF THE TEST 6
8.1. Prior to start the test : 6
8.2. Assay procedure : 6
8.3. Direct reading of the results 7
8.4. Automatic colorimetric reading of the results 7
9. PERFORMANCES OF THE TEST 9
9.1. Clinical sensitivity 9
9.2. Clinical specificity 9
9.3 Repeatability 9
9.4. Interferences 9
9.5. Cross reactivities 10
10. PRECAUTIONS AND WARNINGs 10
10.1 Particular attention 10
10.2. Safety note for reagents containing hazardous substances 11
10.3. Waste disposal 11
11. SYMBOLS USED ON THE LABELS 11

1. INTENDED USE

The SirYus CoViDiag kit is intended for the qualitative determination of IgG class antibodies against SARS-CoV-2 in human serum or plasma to support the diagnosis of COVID-19 infection.
This kit constitutes a supplement to direct pathogen detection.
In addition, serology can be used to collect epidemiological information on the prevalence of SARS-CoV-2.

2. CONTEXT

End of 2019, a novel respiratory disease emerged in the city of Wuhan, Hubei Province of the People's Republic of China, and soon spread rapidly within the country and worldwide. The causative agent was identified as severe acute respiratory syndrome coronavirus 2 (SARS-CoV-2).
The clinical manifestations of SARS-CoV-2-related COVID-19 infection include fever, cough, respiratory problems and fatigue. In most patients the affection shows symptoms of a mild febrile illness with irregular lung infiltrates.

Different methodologies of diagnostic tests are available:

- Molecular tests for the detection of the SARS-CoV-2 coronavirus genome by RT-PCR which is, at present, the only technique for COVID-19 diagnostic recommended,
- Antigenic tests which allow the detection of specific SARS-CoV-2 proteins. Given their poor performance, these antigenic tests are not currently recommended,
- Serological tests that intent to detect specific antibodies (Ab) produced by the organism and directed against SARS-CoV-2.

Few consolidated data are currently available on the immune response against SARS-CoV-2. However, based on the first published studies, a set of observations agree on several aspects of the adaptive humoral immune response, and more particularly on the kinetics of anti-SARS-CoV-2 antibody production.
The IgG production is detectable in symptomatic patients from the second week after onset of symptoms. However, cases have also been reported with later antibody production, beyond the 15th day after the onset of symptoms, and up to 30 days after infection, especially in asymptomatic or paucisymptomatic patients.
Serological tests cannot tell whether the person is contagious or not. This is because seroconversion is not accompanied by a drop in viral titer. There is no established correlation between the production of antibodies and the presence of the infectious virus. In addition, neither the infectious dose of SARS-CoV-2 nor the dose initiating an antibody response is currently known.
Serologic testing can only determine whether a person has produced antibodies in response to infection with the virus, in other words, whether or not that person has triggered an immune response against the virus. But an immune response is not always a proof of protective immunization against further infection with the same virus. To get a protective immunization, it is necessary that the organism produces high titers of antibodies that prevents the action of the virus and in particular its entry into the target cells. We then speak about neutralizing antibodies.
In addition, these important titers of neutralizing antibodies have to be produced over a long period to ensure a protection over the time. However, the target epitopes of neutralizing antibodies have not yet been identified.
Regarding the viral targets used in serological tests to detect IgG, two proteins are frequently used: protein S (spike), the surface protein of the virus allowing interaction (via its RBD domain) and fusion with the target cell and the N protein (core protein, internal to the virus). While the S protein (or its RBD domain) has been described as inducing an earlier response or better correlated with the presence of neutralizing antibodies, the precise neutralizing epitopes have not been characterized.
These epitopes may also depend on the conformation of the protein, in particular as regards the S protein. To date, there is therefore no robust element to promote the use of a precise form or of another of these proteins.
Serological tests are performed on blood samples and are intended to identify patients who have developed immunity to SARS-CoV-2 whether they have been symptomatic or not. Serological tests

could identify in certain circumstances the patients being or having been infected by SARS-CoV-2 or monitor the serological status of exposed persons (healthcare professionals for example).
Finally, these tests can also be useful in collecting epidemiological data related to COVID-19 (actually infected patients, rate of severe forms, etc.) or open perspectives for prognostic data.

3. PRINCIPLE OF THE TEST

The qualitative immunoenzymatic detection of specific antibodies is based on ELISA technology principle. Regarding SirYus CoViDiag kit, 5 different ELISA-like assays are miniaturized and combined.
Microtitration plates wells were printed with 5 differents antigens from SARS-CoV-2 separetly. They are dedicated to bind specific antibodies in the tested samples and therefore deliver 5 different responses in one single assay.

Printed antigens are :

- N protein (Nucleocapside protein, internal to the virus),
- S1 protein (Sub-unit 1 of the Spike protein),
- RBD domain of the S1 protein (Receptor Binding Domain),
- NTD domain of the S1 protein (N-Terminal Domain),
- S2 protein (Sub-unit 2 of the Spike protein).

Diluted sample (sera or plasma) is incubated in a microtitration plate well. After a washing step to remove the sample, a secondary antibody conjugated to an enzyme (HRP) is added. This conjugate binds to antibodies already captured from the sample. A second washing step is dedicated to remove excess of unbound conjugate. A chromogen substrate: tétraméthylbenzidine (TMB), is then used in order to detect the antibody's complexes. Distinguishable individual circular blue precipitates will be visible at the bottom of the well when such complexes are present.
The color intensity depends on the amount of specific antibodies present in the sample.
For results interpretation, the well bottom surfaces can be analysed with bare-eyes or imaged using a colorimetric reader.

4. MATERIAL

4.1 Reagents

SirYus Chips | CVD **12 breakable strips of 8 wells in a microplate** ; each well were printed with 5 different antigens of SARS-CoV-2 ; plates are packed in an aluminium sealed foil.

DB | CVD **Dilution Buffer** : 1 x 120 mL bottle of aqueous buffer for sample dilution ; Ready to use ; orange color; with <0.05% Proclin® 300.

WB | CVD **Wash Buffer** : 2 x 120 mL bottles of aqueous buffer for wells washing; ready to use ; blue color ; with <0.05% Proclin® 300.

CA | CVD **Conjugate Ab :** 1 x 100 µL vial of anti-IgG human conjugate to a HRP peroxydase; to be diluted ; Blue cap ;with <0.05% Proclin® 300.

SU | CVD **Substrate :** 1 x 15 mL bottle of unsoluble chromogen substrate (3,3′,5,5′-Tetramethyl-benzidine) for HRP enzyme; ready to use ; brown bottle.

An instruction for use booklet is provided with the kit.

For hazard and precautionary statements see chapter 10.2
For potential hazardous substances please check the safety data sheet.

4.2 Materials and equipment required

- Incubator 37°C
- Deionised water
- Pipettes to deliver volumes between 10 and 1000 µL
- Vortex tube mixer
- 96 wells microplate plate sticker or cover foil
- Disposable tubes or polypropylene plate

4.3 Optional materials and equipment

- Automatic microtiter plate washer
- Microtiter plate Agitator
- Multi-channels pipette
- Colorimetric reader for biochips

5. STABILITY AND STORAGE

Store the kit at 2...8 °C.
Expiry date is indicated on each composant of the kit.
Expiry date of the kit correspond to the expiry date of the composant with the shortest shelf life.
Performances of the test are depending on expiry date. Do not use reagents and tests after the expiry date.

Sample and conjugate dilutions have to be performed for each assay.

6. REAGENTS PREPARATION

It is very important to bring all reagents and samples to room temperature (20…25 °C) 30 min prior to the test and mix them before starting the test run!

6.1 Test well strips

The wells of the plate/breakable strips were printed with 5 differents antigens from SARS-CoV-2 (fig1). Immediately after removal of the strips, the remaining strips should be resealed in the aluminium foil along with the desiccant supplied.
Remaining non-use strips in sealed foil have to stored at 2...8 °C.

	1	2	3	4	5	6	7	8	9	10
1	CTRL+	CTRL+							CTRL+	CTRL+
2			NTD	NTD	NTD					
3										
4			S1	S1	S1		N			N
5	S2		S1				N	N		N
6	S2		S1	S1	S1		N		N	N
7	S2				S1		N			N
8			S1	S1	S1		N			N
9										
10			RBD	RBD	RBD		Samp Ctrl	Samp Ctrl		
11	CTRL+	CTRL+					Samp Ctrl	Samp Ctrl		CTRL+

CTRL+	Positive control
SampCtrl	Sample control
N	(N) Nucleocapside protein
S1	(S1) Sub-unit 1 of Spike protein
RBD	(RBD) Receptor Binding Domain of Spike 1 protein
NTD	(NTD) N-Terminal Domain of Spike 1 protein
S2	(S2) Sub-unit 2 of Spike protein

Figure 1 *– Antigens layout of each wells of the SirYus Covidiag microplate*

6.2 Washing buffer

The Wash Buffer WB | CVD is stable at 2-8 °C. In case salt crystals appear in the bottle, warm up the solution to 37 °C e.g. in a water bath. Mix well before use.
The Kit includes 2 x 120 mL of Wash Buffer WB | CVD. Current procedure requires 120 mL. In case of automatic washer use, do not use more than 120 mL to prime the system.

6.3 Conjugate

Conjugate has to be diluted to 6:1000 in the Dilution Buffer DB | CVD.
For a whole plate, we recommend to prepare 10 mL of conjugate diluted solution.
To prepare 10 mL of conjugate diluted solution, add 60 µL of Conjugate Antibody CA | CVD to 10 mL of Dilution Buffer DB | CVD.

6.4 Substrate solution

The solution SU | CVD is ready to use and has to be stored at 2-8 °C protected from the light.
The solution should be colourless or could have a slight blue tinge. If the substrate turns into blue, it may have been contaminated and should be thrown away.

7. SAMPLES COLLECTION AND PREPARATION

7.1 Samples

Use human serum or plasma (citrate, heparin) samples with this assay.
If the assay is performed within 5 days after sample collection, the samples should be kept at 2...8 °C; otherwise they should be aliquoted and stored deep-frozen (-70…-20 °C). If samples are stored frozen, mix thawed samples before testing. Avoid repeated freezing and thawing.
Heat inactivation of samples is not recommended.

7.2 Sample dilution

All samples should be diluted 1+100 with Dilution Buffer DB | CVD. We recommend to dispense 10 µL sample and 1 mL Dilution Buffer DB | CVD into tubes or polypropylene plate to obtain a 1+100 dilution and thoroughly mix before incubation.

8. PROCEDURE OF THE TEST

Please read the instruction for use carefully before performing the assay.

Result reliability depends on strict adherence to the instruction for use as described. The following test procedure is only validated for manual procedure. If performing the test on ELISA automatic systems we recommend to perform a whole internal validation process.

8.1. Prior to start the test :

- the distribution and identification plan for all samples should be carefully established on the plate layout supplied in the kit (Annex 1).
- Select the right quantity of strips needed. Let the non used strips in the foil with dessicant for further use.
- Perform all assay steps in the order given and without any delays. Avoid any drying of the wells between steps.
- A clean, disposable tip should be used for dispensing each standard/control and sample.
- Adjust the incubator to 37 ± 1 °C.
- If you are using an automatic plate washer, prime the system. Do not use more than 120 mL of Wash Buffer to prime the washing system.

8.2. Assay procedure :

1. Dispense 100 µL of diluted samples in their respective wells (cf chapter 7.2).
2. Cover the wells with a sticker or cover foil (not provided in the kit).
3. Incubate for 1 hour at 37°C (Plate shaker can be used at 300 rpm).
4. When incubation has been completed, remove the foil, aspirate the content of the wells and wash each well three times with 200 µL of Washing Buffer lavage WB | CVD.
   Avoid overflows from the reaction wells. The interval between washing and aspiration should be >5sec **AND NOT EXCEED 5 MIN**. At the end carefully remove remaining fluid by tapping strips on tissue paper prior to the next step!
   Note: Washing is important! Insufficient washing results in poor precision and false results.
5. Dispense 100 µL of diluted conjugate (cf chapter 6.3) in all the wells.
6. Incubate for 1 hour at 37°C. Keep the plate away from the light.
7. Repeat step 4.

8. Dispense 50 µL of Substrate SU | CVD in all the wells.
9. Incubate for exactly 15 minutes at room temperature (20-25 °C) in the dark. Blue colour occurs due to an enzymatic reaction.
10. At the end of incubation, remove any remaining fluids by tapping strips upside down on tissue paper. Dispense 200 µL of deionised water and then remove the liquid. Repeat 3 times.
    After the 3 cycles, tap the strips on tissue paper.
11. Wait for 15 min at 37°C for drying. Keep the plate away from the light.
12. Read the tests.

8.3. Direct reading of the results

Direct reading of the wells leads to get kind of pictures presented in figure 2.

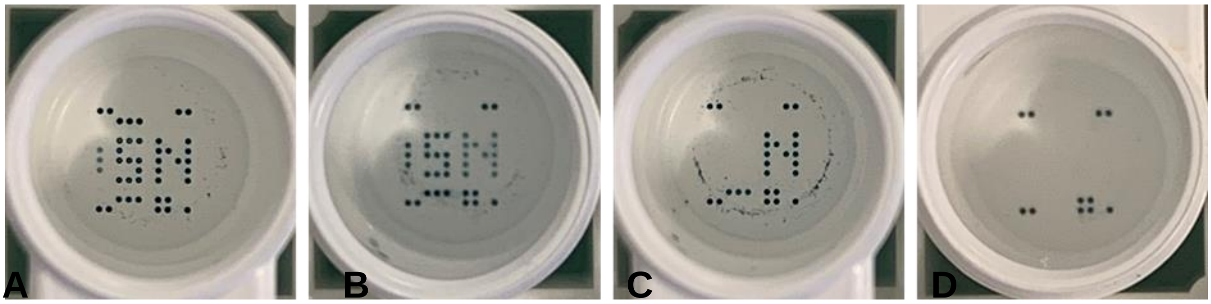

***Figure 2 – Examples of** pictures of the wells after samples testing.*

By using the direct reading sheet (Annex 2), check the boxes when control spots, N and/or S are visible:

- 7 positive controls spots have to be clearly visible. They are validating the testing process. If more than 2 spots are missing, the test can not be validated.
- 4 Sample controls spots (bottom right) have to be clearly visible. They are validating the sample addition. If more than 2 spots are missing, the test can not be validated
- Spots arrayed as a « N » shape are dedicated to the detection of anti-Nucléocapside antibodies. As soon as a « N » is visible, anti-N antibodies are present : Image A, B and C.
- Spots arrayed as a « S » shape are dedicated to the detection of anti-Spike1 antibodies. As soon as a « S » is visible, anti-S1 antibodies are present : Image A and B.

A patient would be seroconverted as soon as N and/or S will be visible.

8.4. Automatic colorimetric reading of the results

We recommend to follow the user guide provided by reader's manufacturer in order to read the tests and extract data. The test interpretation is performed from data provided by the reader.

The following interpretation process for SirYus CoviDiag picture analysis is described for a colorimetric biochips reader SciReader (Scienion, Berlin, Germany) as example:

For further interpretation steps, use the mean of the net signals from replicates spots for each antigens.
Net signals are corresponding to signal measured on each spot and substracted by background (back ground signal around the antigen spot).

From Mean net signals, define patient status (NEG / BORDER / POS) for each antigen of the test:

Antigen / (A.U.)	NEG	BORDER	POS
**Nucléocapside**	0 - 15	15 – 30	> 30
**Spike S1**	0 - 10	10 – 20	> 20
**Spike S1 – RBD**	0 - 10	10 – 20	> 20
**Spike S1 – NTD**	0 - 10	10 – 20	> 20
**Spike S2**	0 - 15	15 – 30	> 30

The cut off values were defined from results obtained by testing 200 plasmas and serums from pre-pandemic healthy subjects (before June 2019).

Interpretation and positivities of samples were done as following :

- **Technical validation of the test** : a test will be validated as soon as the mean of net signals from positive controls: CTRL+ and Samp Ctrl will be above 10 A.U.

- **Sample identified as Positive :**
  - *S1 and/or RBD and/or NTD is POS*
    OR
  - *S2 and/or N is > 40 A.U.*
    OR
  - *S2 and/or N is POS and S1 and/or RBD and/or NTD and/or S2 and/or N is BORDER.*
    OR
  - *S2 and/or N is BORDER and S1 and/or RBD and/or NTD and/or S2 and/or N is BORDER.*

- **Samples identified as Borderline :**
  - *S2 and/or N is POS*
    OR
  - *S1 and/or RBD and/or NTD is BORDER.*

- **Samples identified as Negative :**
  All others case not listed above.

9. PERFORMANCES OF THE TEST

9.1. Clinical sensitivity

Clinical sensitivity is measured as the ability of the test to detect anti-SARS-CoV-2 antibodies, in sera and plasmas from patient already identified as affected by COVID-19.
106 patients already tested as positive by RT-PCR were tested with the SirYus CoViDiag kit.

Days between onset of symptoms and sample collection	Samples number	Direct reading				Automatic reading			
		Positive	Border.	Negative	Sensitivity	Positive	Border.	Negative	Sensitivity
< 8 days	9	5	1	3	56%	7	1	1	78%
8 to 14 days	40	34	2	4	85%	36	0	4	90%
> 14 days	57	56	0	1	98%	57	0	0	100%

Immune response appears at least 7 days after symptoms onset (Zhao et al. 2020).
In this clinical study, 90/97 patients collected more than 8 days after symptoms onset have been tested as Positive by **direct reading**, giving **92,8% of sensitivity**.
With **Automatic reading**, 93/97 patients were tested as positive.**The sensitivity reaches 95,9%**.

9.2. Clinical specificity

Clinical specificity is corresponding to the ability to get negative results when testing healthy subjects. This would insure the test is not detecting any unspecific reaction.
240 samples from healthy subject collected before June 2019 were tested.

Number of negative samples	Direct reading				Automatic reading			
	Positive	Border.	Negative	Specificity	Positive	Border.	Negative	Specificity
240	3	4	233	98,8%	1	0	239	99,6%

In this clinical study, 237/240 healthy subjects were not tested as positive by **direct reading**, giving **98,8 % of specificity**.
With **automatic reading**, 239/240 pre pandemic samples were not tested as positives and the **specificity reaches 99,6%.**

9.3 Repeatability

Repeatability is measured by performing 46 assays at least on the same sample. 4 samples were used.

	Nucleocapside								Spike S1							
	Sample. #1		Sample. #2		Sample. #3		Sample. #4		Sample. #1		Sample. #2		Sample. #3		Sample. #4	
	n = 46		*n = 46*		*n = 48*		*n = 48*		*n = 46*		*n = 46*		*n = 48*		*n = 48*	
**Mean signal**	**84,34**		**56,00**		**76,65**		**0,11**		**72,33**		**70,38**		**67,97**		**0,06**	
Min. / Max.	*78,3*	*93,1*	*46,5*	*61,9*	*72,3*	*82,5*	*0,009*	*0,514*	*68,7*	*77,6*	*65,8*	*75,1*	*64,2*	*74,4*	*0,004*	*0,187*
**Interprétation**	Positive		Positive		Positive		Negative		Positive		Positive		Positive		Negative	
%Positive	100%		100%		100%		0%		100%		100%		100%		0%	
%Questionable	0%		0%		0%		0%		0%		0%		0%		0%	
%negative	0%		0%		0%		100%		0%		0%		0%		100%	
	SD	***CV***	SD	***CV***	SD	***CV***	SD	***CV***	SD	***CV***	SD	***CV***	SD	***CV***	SD	***CV***
**Repeatability**	3,474	***4,12%***	2,976	***5,31%***	2,657	***3,47%***	0,102	***91,6%***	1,680	***2,32%***	2,076	***2,95%***	2,361	***3,47%***	0,039	***66,7%***

On the whole study, no diagnostic switch is observed.
Signals from expected negative antigens are very low. This would explain higher CV. Anyway, this higher CV are not representative of the repeatability of the test.
The average repeatability is 5.8 %.

9.4. Interferences

Three different samples (2 samples expected as positive and 1 sample expected as negative) were tested to assess potential interferences with different substances listed below.

Interferent	Concentration tested
Albumin	60 mg/mL
Bilirubine	0,4 mg/mL
Cholestérol	4 mg/mL
Hémoglobine	10 mg/mL
Triglycérides	15 mg/mL

For the tested concentrations, no significative interferences were observed.

9.5. Cross reactivities

57 samples presenting antibodies against different pathogens (included antibodies against different respiratory pathogens) were tested in order to assess the analytical specificity and cross reactivity of the assay.

Pathogen	Samples number	CoViDiag test (IgG)	
		POS Nber	NEG Nber
hCoV 229E	12	0	12
hCoV OC43	8	0	8
hCoV HKU1	1	0	1
hCoV NL63	4	0	4
Grippe A&B	5	0	5
Legionella	2	0	2
Haemophilus	3	0	3
Rougeole	2	0	2
Parvovirus B19	3	0	3
CMV	3	0	3
Syphillis	3	0	3
Toxoplasmose	3	0	3
HIV	1	0	1
HCV	4	0	4
HBs	3	0	3
**TOTAL**	**57**	**0**	**57**

Results presented in the table were acquired by direct reading and automatic reading as well.
No cross reactivity has been observed.

10. PRECAUTIONS AND WARNINGs

10.1 Particular attention

- In compliance with article 1 paragraph 2b European directive 98/79/EC the use of the in vitro diagnostic medical devices is intended by the manufacturer to secure suitability, performances and safety of the product. Therefore the test procedure, the information, the precautions and warnings in the instructions for use have to be strictly followed. The use of the test kits with analyzers and similar equipment has to be validated. Any change in design, composition and test procedure as well as for any use in combination with other products not approved by the manufacturer is not authorized; the user himself is responsible for such changes. The manufacturer is not liable for false results and

incidents for these reasons. The manufacturer is not liable for any results by visual analysis of the patient samples.

- The present kit is dedicated to in vitro diagnostic use.
- SirYus CoViDiag is only designed for qualified personnel who are familiar with good laboratory practices.
- All materials of human or animal origin should be regarded and handled as potentially infectious.
- Do not interchange reagents or Microtiterplate strips of different production lots.
- No reagents of other manufacturers should be used along with reagents of this test kit.
- Do not use reagents after expiry dates stated on the label.
- Use only clean pipette tips, dispensers, and lab ware.
- Do not interchange screw caps of reagent vials to avoid cross-contamination.
- Close reagent vials tightly immediately after use to avoid evaporation and microbial contamination.
- After first opening and subsequent storage check conjugate and standard/control vials for microbial contamination prior to further use.
- To avoid cross-contamination and falsely elevated results, pipette patient samples and dispense reagents without splashing into the wells.
- Use only fresh prepared diluted conjugate solution.
- Before any use, check the substrate solution. If the substrate turns blue, it may have been contaminated and should be thrown away.

10.2. Safety note for reagents containing hazardous substances

Reagents may contain <0.05 % of ProClin 300 (See chapter 4.1).
The chromogen substrate of the enzyme is 3,3′,5,5′-Tetramethyl-benzidine – TMB.

Therefore, the following hazard and precautionary statements apply.

10.3. Waste disposal

Residues of chemicals and preparations are generally considered as hazardous waste. The disposal of this kind of waste is regulated through national and regional laws and regulations. Contact your local authorities or waste management companies which will give advice on how to dispose hazardous waste.

11. SYMBOLS USED ON THE LABELS

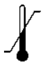	Storage temperature
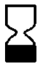	Expiry date
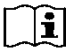	Read IFU prior to use the kit
SC | CVD	SirYus-Chip CoViDiag
WB | CVD	Wash Buffer CoViDiag

DB | CVD	Diluant Buffer CoViDiag
CA | CVD	anti-human IgG Conjugate – HRP (horseradish peroxydase)
SU | CVD	Chromogenic substrate of the HRP enzyme
IVD	For In Vitro Diagnostic use
LOT	Batch Number
REF	Catalogue Reference

Annexe 1 – Plate layout

H	G	F	E	D	C	B	A	
								1
								2
								3
								4
								5
								6
								7
								8
								9
								10
								11
								12

Annexe 2 – Direct reading sheet

Operator name

	CTRL+	Ech.	N	S1
Wells #A1				
Wells #A2				
Wells #A3				
Wells #A4				
Wells #A5				
Wells #A6				
Wells #A7				
Wells #A8				
Wells #A9				
Wells #A10				
Wells #A11				
Wells #A12				
Wells #B1				
Wells #B2				
Wells #B3				
Wells #B4				
Wells #B5				
Wells #B6				
Wells #B7				
Wells #B8				
Wells #B9				
Wells #B10				
Wells #B11				
Wells #B12				
Wells #C1				
Wells #C2				
Wells #C3				
Wells #C4				
Wells #C5				
Wells #C6				
Wells #C7				
Wells #C8				
Wells #C9				
Wells #C10				
Wells #C11				
Wells #C12				
Wells #D1				
Wells #D2				
Wells #D3				
Wells #D4				
Wells #D5				
Wells #D6				
Wells #D7				
Wells #D8				
Wells #D9				
Wells #D10				
Wells #D11				
Wells #D12				

#Plate

	CTRL+	Ech.	N	S1
Wells #E1				
Wells #E2				
Wells #E3				
Wells #E4				
Wells #E5				
Wells #E6				
Wells #E7				
Wells #E8				
Wells #E9				
Wells #E10				
Wells #E11				
Wells #E12				
Wells #F1				
Wells #F2				
Wells #F3				
Wells #F4				
Wells #F5				
Wells #F6				
Wells #F7				
Wells #F8				
Wells #F9				
Wells #F10				
Wells #F11				
Wells #F12				
Wells #G1				
Wells #G2				
Wells #G3				
Wells #G4				
Wells #G5				
Wells #G6				
Wells #G7				
Wells #G8				
Wells #G9				
Wells #G10				
Wells #G11				
Wells #G12				
Wells #H1				
Wells #H2				
Wells #H3				
Wells #H4				
Wells #H5				
Wells #H6				
Wells #H7				
Wells #H8				
Wells #H9				
Wells #H10				
Wells #H11				
Wells #H12				
